# Alzheimer's clinical research data via R packages: The alzverse

**DOI:** 10.1002/alz.71152

**Published:** 2026-01-29

**Authors:** Michael C. Donohue, Kedir Hussen, Oliver Langford, Richard Gallardo, Gustavo Jimenez‐Maggiora, Paul S. Aisen, for the Alzheimer's Disease Neuroimaging Initiative

**Affiliations:** ^1^ Epstein Family Alzheimer's Therapeutic Research Institute University of Southern California San Diego California USA

**Keywords:** Alzheimer's, clinical trial, open data, R

## Abstract

**INTRODUCTION:**

Sharing clinical research data is essential for advancing Alzheimer's disease (AD) research, yet challenges in accessibility, standardization, documentation, usability, and reproducibility persist.

**METHODS:**

We developed R data packages to streamline access to curated datasets from key AD studies. A4LEARN includes data from the Anti‐Amyloid Treatment in Asymptomatic Alzheimer's (A4) randomized trial and its companion observational study, the Longitudinal Evaluation of Amyloid Risk and Neurodegeneration (LEARN). ADNIMERGE2 contains curated data from the Alzheimer's Disease Neuroimaging Initiative (ADNI), a longitudinal biomarker and imaging study.

**RESULTS:**

These packages bundle data, documentation, and reproducible analysis vignettes into portable, analysis‐ready formats that can be installed and used within R. We also introduce the alzverse package, which applies a common data standard to integrate study‐specific packages and facilitate meta‐analyses.

**DISCUSSION:**

By promoting collaboration, transparency, and reproducibility, R data packages provide a scalable framework to accelerate AD clinical research.

**Highlights:**

- R packages enable access to curated Alzheimer's clinical study datasets.
- A4LEARN and ADNIMERGE2 provide portable, analysis‐ready data resources.
- R packages integrate data, documentation, and reproducible analysis vignettes.
- alzverse unifies study packages via common standards to support meta‐analyses.
- Tools promote transparency, collaboration, and reproducibility in Alzheimer's disease (AD) research

## 1 BACKGROUND

Alzheimer's disease (AD) is among the most important neurodegenerative diseases worldwide. The Alzheimer's Disease Neuroimaging Initiative (ADNI)^1^ and similar projects have accumulated vast quantities of clinical, neuroimaging, and biomarker data, creating opportunities for scientific advances in the understanding and treatment of AD. ADNI data have supported more than 6000 scientific publications.^1^

Availability of such data is on the rise, due in part to data‐sharing mandates from funders like the National Institutes of Health. However, it often takes considerable time and effort for researchers to gain sufficient familiarity with the data to produce meaningful analyses. Learning curves can be steep due to inadequate or hard‐to‐locate documentation and example analysis code.

Open‐source software solutions, particularly in the form of R packages,^2^, ^3^ offer significant potential to address these barriers. The R programming language is widely used in biostatistics, machine learning, and clinical research. R packages are a well‐known means for distributing cutting‐edge statistical software and documentation, and they often include data and analysis vignettes that demonstrate how the methods can be applied. Importantly, R packages can also be an effective tool to share data itself. For example, the authors have maintained the ADNIMERGE R data package since 2013. It has been cited in about 250 articles^1^ and has inspired related projects, such as the ANMERGE package of AddNeuroMed Consortium data.^4^ Similarly, Vuorre and Crump^5^ demonstrated the utility of R packages for sharing data and analysis code from psychological experiments.

We discuss how R packages can also facilitate easy access, harmonization, and analysis of larger clinical datasets from AD studies. These packages are built with the goal of providing an audit trail of derived data provenance, supporting reproducible research, and leveraging outstanding R tools for unit testing and validation,^6^ websites,^7^ and regulatory submissions.^8^

In this paper, we discuss the advantages of using R packages to share large clinical study datasets, and provide two new examples: the A4LEARN packages which includes data from a randomized trial (the Anti‐Amyloid Treatment in Asymptomatic Alzheimer's [A4] study)^9^ and its companion observational study of biomarker negative individuals (the Longitudinal Evaluation of Amyloid Risk and Neurodegeneration [LEARN] study)^10^; and the ADNIMERGE2 package, which includes latest data from the Alzheimer's Disease Neuroimaging Initiative (ADNI).^1^ We also introduce the alzverse metadata package,^11^ which demonstrates how ADNIMERGE2, A4LEARN, and other R data packages can be combined to facilitate meta‐analyses.

## 2 METHODS

### 2.1 Reproducibility, portability, and documentation

The most important advantage of using R data packages for sharing clinical research data is that it facilitates reproducible research. R is widely available and free to download,^2^ commonly used in statistics courses, and has active development communities including academic statisticians, pharmaceutical statisticians, and commercial enterprises such as Posit's RStudio Integrated Development Environment (IDE).

**RESEARCH IN CONTEXT**

1. **Systematic review**: We reviewed published literature on data sharing in Alzheimer's disease (AD) clinical research, focusing on reproducibility, data accessibility, and computational infrastructure. Relevant resources included prior work on Alzheimer's Disease Neuroimaging Initiative (ADNI), Anti‐Amyloid Treatment in Asymptomatic Alzheimer's/Longitudinal Evaluation of Amyloid Risk and Neurodegeneration (A4LEARN), and broader efforts in reproducible research and R‐based data curation.
2. **Interpretation**: Our work introduces R data packages (A4LEARN, ADNIMERGE2, and alzverse) that address barriers to accessibility and reproducibility by bundling curated AD clinical datasets, documentation, and reproducible analyses into portable, analysis‐ready formats. These tools expand the infrastructure available for secondary use of AD trial and observational data.
3. **Future directions**: Future research should extend these methods to additional Alzheimer's Clinical Trials Consortium (ACTC) and Alzheimer's Therapeutic Research Institute (ATRI) ‐managed trials, evaluate integration with regulatory and Clinical Data Interchange Standards Consortium (CDISC) data standards, and test scalability for meta‐analyses across studies. Broader adoption will require active development and user communities.

All R packages include a manual that details the functions and datasets in a standardized format. This content is linked to the R object's name, is readily accessible to the R user (e.g., by typing “?t.test”, or “?cars”), and can be browsed within the IDE. This is a substantial improvement over the typical case in which relevant data documentation might be scattered across unlinked documents or data dictionary spreadsheets. R packages also typically provide analysis “vignettes”, which demonstrate how functions can be applied to available datasets to produce analysis results as tables or figures. These are also linked and can be browsed within the IDE. Our A4LEARN package contains a vignette to exactly reproduce key findings of the published manuscript reporting the trial results.^9^ This code can be used by outside researchers to jump‐start their own inquiries and help ensure the data is being used correctly and efficiently in a manner consistent with the intentions of the study team.

The R package bundle of data, R functions, documentation, and vignettes is made portable as an efficiently compressed file which can be installed on any machine running R. Data files within the package are also efficiently compressed using R's .RData file format. These .RData files can be read by SPSS, Stata, and SAS, allowing researchers to access and analyze the data using a variety of statistical software beyond R. Another advantage of .RData compared to tabular text files (e.g., .csv files), is that they can utilize R object classes such as dates and factor variables, eliminating the need to process and annotate data prior to analysis.

The pkgdown R package ^7^ makes it trivial to export documentation and vignettes as a website, and integrates well with code repositories such as GitHub. These pkgdown websites are searchable and provide access to documentation and examples for researchers, even if they do not use R. See A4LEARN and ADNIMERGE2 project documentation on the Alzheimer's Therapeutic Research Institute (ATRI) GitHub site.^12^, ^13^

### 2.2 Standardized and efficient workflow and testing

Questions often arise about the original source of data or how derived variables were determined. Therefore, it is crucial to preserve a record of data provenance. This is important whenever reproducible research is a goal, and particularly for submissions to regulators, such as the United States Food and Drug Administration (FDA). The standardized R package structure and build workflow make the data provenance transparent. R packages also have a standardized framework for testing^6^ and tools for “assertive” programming to verify assumptions about the data.^14^

The R package structure and workflow have been well‐documented.^3^ Here, we briefly review the structure while highlighting some key aspects in the context of the clinical research data.

**data‐raw**. The data‐raw directory is intended to contain raw data and code to import and process raw data and store .Rdata files in the data directory. Raw data can be preserved in the package with minimal manipulation, or it can be processed by attaching metadata (variable labels and units) and ensuring factors and dates are stored as the correct object class. Data dictionary spreadsheets can be parsed to provide content for manual pages.^15^

**vignettes**. The vignettes directory contains analysis demonstrations, typically as Rmarkdown (.Rmd) files.^16^ We prefer to create derived datasets and variables in the form of a vignette as well, so that derivations are easily accessible to researchers within the IDE. These vignettes can include assertive programming to ensure data conforms to expectations.^14^ ADNIMERGE2 contains vignettes that use pharmaverse workflows to derive Clinical Data Interchange Standards Consortium (CDISC) Analysis Data Model (ADaM) datasets.^17^ The ADaM format is part of the CDISC standards used in clinical trials to create analysis‐ready datasets that are consistent, traceable, and regulatory‐compliant. ADaM specifies standard structures and metadata for datasets used in statistical analysis and regulatory submissions (e.g., to the FDA), ensuring traceability from raw data to analysis datasets and ultimately to tables, listings, and figures. Common ADaM datasets include: ADSL (Subject‐Level Analysis Dataset with one record per subject; demographics, treatment, key flags), ADAE (Adverse Events Analysis Dataset), ADLB (Laboratory data Analysis Dataset), ADQS (Questionnaire data with one record per subject, time point, and outcome type).

**R**. The R directory contains .R files with code defining R functions and their manual content.^13^ This directory can store scoring functions, which might be necessary to derive scores from item‐level data from psychometric assessments, for example.

**testthat**. The testthat directory includes automated tests that are checked when the package is built. Critically important code that merits replication by independent programmers can be tested here to ensure equivalent results with real or test data.

**reports**. Report code that is not desired as a vignette can be stored in a separate directory for general reports. Of note, rmarkdown supports “parameterized reports”, which can produce different output depending on the supplied parameter(s). Clinical trials like A4 often include several outcomes collected on the same schedule and analyzed with the same approach. Instead of writing identical code for several outcomes, one generic parameterized rmarkdown file can produce all these reports. Clinical trial outcomes are often aggregated into one long dataset with a row for each subject, time point, and outcome (see ADQS in the A4LEARN package). The parameterized report can filter this long dataset for the desired outcome and analyze only that outcome. Furthermore, using R parallel programming tools (e.g., reference 18), these reports can be produced in parallel. In the case of the A4 trial readout, once data from the blinded phase was locked and unblinded, it only took about 30 minutes to build the final data package and render all planned analysis reports and summary slide decks. Prior to data lock, code and output were tested and[Table alz71152-tbl-0001] reviewed using pseudo‐arm codes.

## 3 RESULTS

### 3.1 The ADNIMERGE2 and A4LEARN R data packages

The primary data sources for the packages are derived from the ADNI and the A4 and LEARN companion studies.^19^ ADNI data used in the preparation of this article and the ADNIMERGE2 package were obtained from the ADNI database.^20^ The ADNI was launched in 2003 as a public‐private partnership, led by Principal Investigator Michael W. Weiner, MD. The primary goal of ADNI has been to test whether serial magnetic resonance imaging (MRI), positron emission tomography (PET), other biological markers, and clinical and neuropsychological assessment can be combined to measure the progression of mild cognitive impairment (MCI) and early AD. ADNI is a flagship resource that set the standard for open data sharing, making it a cornerstone for reproducible Alzheimer's research. ADNI is an invaluable resource for biomarker validation, disease progression modeling, imaging‑derived phenotypes, genotype–phenotype associations, and methods for multimodal harmonization across phases.

A4 was a Phase 3, double‑blind, placebo‑controlled secondary prevention trial enrolling cognitively unimpaired adults (65‐85) with elevated amyloid on PET. Participants received solanezumab, which targets soluble Aβ, or a placebo, for approximately 4.5 years. Top‑line results show no slowing of cognitive decline.^9^ The A4 Study began enrolling in 2014, was completed on June 8, 2023, and results were published on July 17, 2023. Participants in A4 were offered open‐label solanezumab after completing the double‐blind phase, and data from this phase are also available in the shared database. LEARN was A4's companion observational cohort: it followed individuals who screen‑failed A4 due to absent/equivocal amyloid elevation but otherwise met eligibility.^10^ It matches A4 procedures (clinical/cognitive every 24 weeks) and was designed to characterize cognitive trajectories independent of elevated amyloid and to serve as a critical comparison group for A4 and future trials. A4 and LEARN data can be used to assess the consequences of preclinical amyloid elevation on cognition and biomarkers; assess relationships among amyloid burden, subjective/partner‑reported assessments of decline, and performance on cognitive tests; and design the next generation prevention trials.

The screening and baseline data from A4 and LEARN were released in December 2018, a year after enrollment concluded. All A4 and LEARN data, including screening, blinded treatment phase, and open‐label extension phase, were released in July 2024, about a year after data lock.^19^ A4 and LEARN data is available from the A4 and LEARN Study Data Portal.^21^

To ensure usability and consistency, we curated the datasets by mapping variables to standardized terminologies (e.g., CDISC ADaM), handling missing data through imputation techniques, and deriving key analysis variables. A4LEARN and ADNIMERGE2 were developed using best practices in R package development, including:

1. R Package Architecture: Each package is modular, supporting various stages of data analysis, from raw data processing to the generation of regulatory‐compliant datasets.
2. Data Standardization: The packages support standardization of clinical data, with built‐in functions to harmonize variable formats, handle missing values, and generate standardized metadata.
3. Reproducibility: Built‐in vignettes and examples guide users through the installation, data loading, and analysis processes. The packages integrate with tools like renv and Docker to facilitate reproducibility in different computing environments.

The R package framework facilitates the creation of ADaM datasets, which are the gold standard for statistical analysis in clinical trials. The admiral package^22^ is used to generate ADaM datasets for ADNIMERGE2. Other pharmaverse tools can be used to create regulatory‐compliant tables, listings, and figures. R code to produce the example summaries below is included in the Appendix.

**FIGURE 1:**
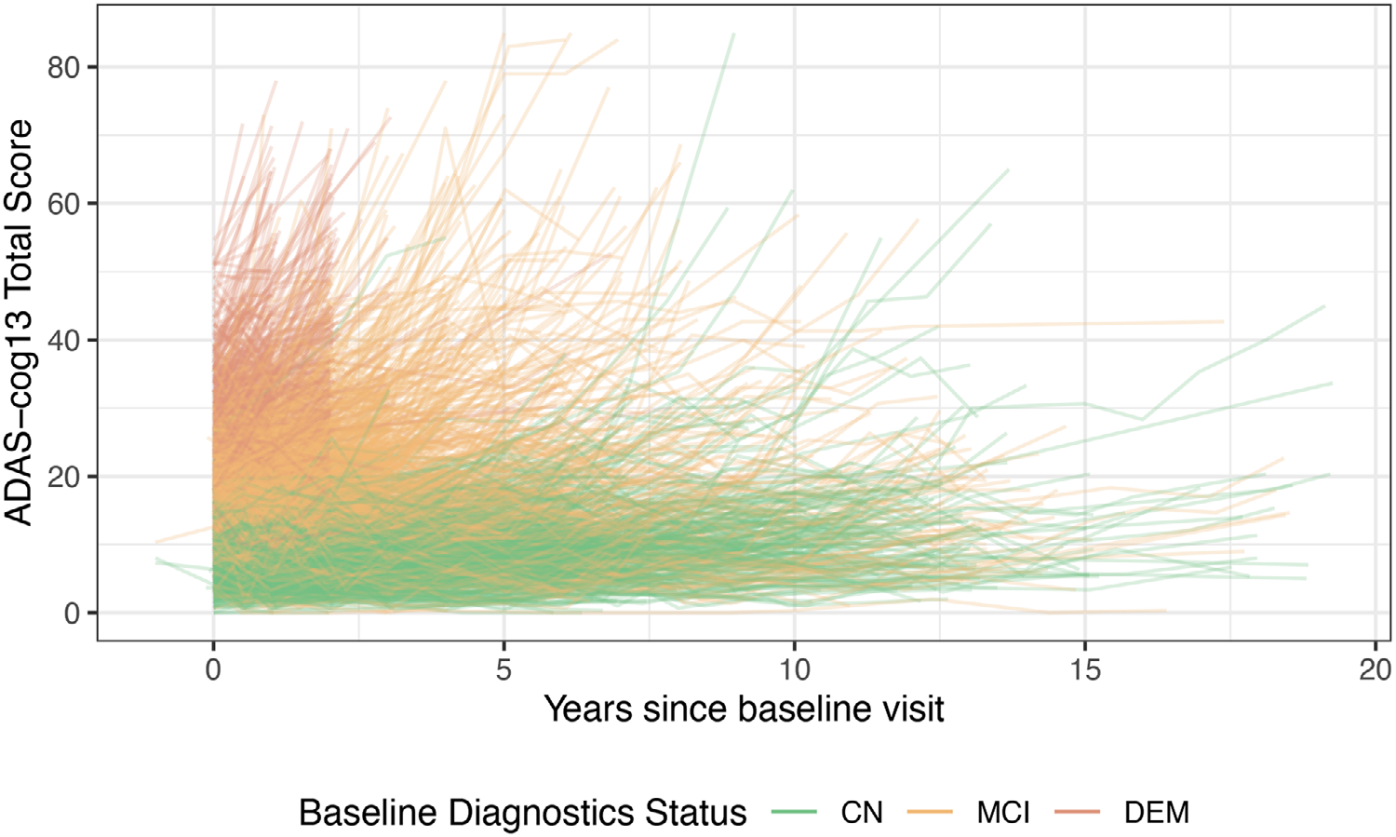
Spaghetti plot of ADAS‐Cog13 scores in ADNI by baseline clinical diagnosis. ADAS‐Cog13, Alzheimer's Disease Assessment Scale‐Cognitive Subscale; ADNI, Alzheimer's Disease Neuroimaging Initiative.

**FIGURE 2:**
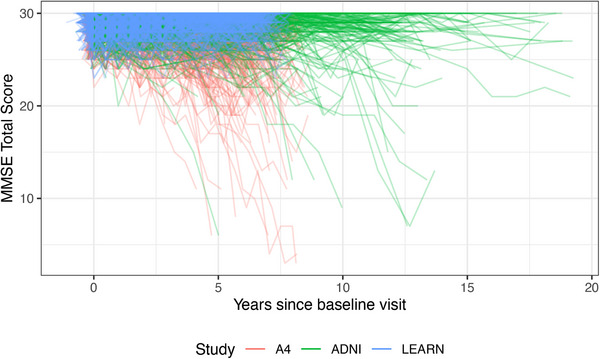
Spaghetti plot of MMSE scores in ADNI Cognitively Normal, A4, and LEARN. A4, Anti‐Amyloid Treatment in Asymptomatic Alzheimer's study; ADNI, Alzheimer's Disease Neuroimaging Initiative; LEARN, Longitudinal Evaluation of Amyloid Risk and Neurodegeneration; MMSE, Mini‐Mental State Examination.

The ADNIMERGE2 package was built using pharmaverse tools. It can be downloaded from the ADNI website.^20^ Below are examples of some basic summaries of participant characteristics by phase, or wave, of ADNI that can be created using the ADSL table (Table 1) and a spaghetti plot of Alzheimer's Disease Assessment Scale‐Cognitive Subscale (ADAS‐Cog) scores using the ADQS table (Figure 1). ADNIMERGE2 is updated quarterly, and the date that the data were sourced is available from the variable ADNIMERGE2::DATA_DOWNLOADED_DATE. Similarly, the A4LEARN package data can be easily summarized using its SUBJINFO table (Table 2). Finally, data from all three studies can be easily summarized using the meta ADQS table of the alzverse package (Figure 2).

**TABLE 1 alz71152-tbl-0001:** ADNI: Subject characteristics by study phase.

Characteristic	ADNI1 *N* = 819^*^	ADNIGO *N* = 131^*^	ADNI2 *N* = 790^*^	ADNI3 *N* = 696^*^	ADNI4 *N* = 596^*^	Overall *N* = 3032^*^
Age (in years)						
Mean (SD)	75.2 (6.8)	71.6 (7.9)	72.7 (7.2)	70.7 (7.4)	69.1 (7.6)	72.2 (7.6)
(Missing)	0	0	0	0	1	1
Sex, *n* (%)						
Female	342 (42%)	60 (46%)	379 (48%)	381 (55%)	367 (62%)	1529 (50%)
Male	477 (58%)	71 (54%)	411 (52%)	315 (45%)	229 (38%)	1503 (50%)
Education						
Mean (SD)	15.5 (3.0)	15.8 (2.7)	16.3 (2.6)	16.4 (2.3)	15.8 (2.9)	16.0 (2.8)
(Missing)	1	0	0	0	2	3
Race, n (%)						
American‐Indian or Alaskan native	1 (0.1%)	1 (0.8%)	1 (0.1%)	2 (0.3%)	4 (0.7%)	9 (0.3%)
Asian	14 (1.7%)	1 (0.8%)	14 (1.8%)	29 (4.2%)	49 (8.2%)	107 (3.5%)
Black or African‐American	39 (4.8%)	4 (3.1%)	34 (4.3%)	105 (15%)	207 (35%)	389 (13%)
Native Hawaiian or Other Pacific Islander	0 (0%)	0 (0%)	2 (0.3%)	1 (0.1%)	2 (0.3%)	5 (0.2%)
Other Pacific Islander	0 (0%)	0 (0%)	0 (0%)	0 (0%)	0 (0%)	0 (0%)
White	762 (93%)	118 (90%)	728 (92%)	537 (77%)	297 (50%)	2442 (81%)
More than one race	3 (0.4%)	5 (3.8%)	10 (1.3%)	13 (1.9%)	26 (4.4%)	57 (1.9%)
Unknown	0 (0%)	2 (1.5%)	1 (0.1%)	9 (1.3%)	11 (1.8%)	23 (0.8%)
Ethnicity, *n* (%)						
Hispanic or Latino	19 (2.3%)	8 (6.1%)	31 (3.9%)	58 (8.3%)	78 (13%)	194 (6.4%)
Not Hispanic or Latino	794 (97%)	122 (93%)	755 (96%)	637 (92%)	516 (87%)	2824 (93%)
Unknown	6 (0.7%)	1 (0.8%)	4 (0.5%)	1 (0.1%)	2 (0.3%)	14 (0.5%)
Baseline Diagnostics Status, *n* (%)						
CN	229 (28%)	1 (0.8%)	295 (37%)	378 (54%)	312 (52%)	1215 (40%)
MCI	397 (48%)	128 (99%)	344 (44%)	244 (35%)	225 (38%)	1338 (44%)
DEM	193 (24%)	0 (0%)	151 (19%)	74 (11%)	59 (9.9%)	477 (16%)
(Missing)	0	2	0	0	0	2
APOE genotype, *n* (%)						
ε2/ε2	2 (0.2%)	0 (0%)	3 (0.4%)	1 (0.1%)	3 (1.2%)	9 (0.3%)
ε2/ε3	53 (6.5%)	9 (7.0%)	66 (8.5%)	52 (7.7%)	18 (7.1%)	198 (7.5%)
ε2/ε4	18 (2.2%)	2 (1.6%)	14 (1.8%)	17 (2.5%)	11 (4.3%)	62 (2.3%)
ε3/ε3	363 (44%)	67 (52%)	352 (45%)	347 (51%)	114 (45%)	1243 (47%)
ε3/ε4	295 (36%)	42 (33%)	269 (35%)	205 (30%)	85 (33%)	896 (34%)
ε4/ε4	88 (11%)	8 (6.3%)	75 (9.6%)	53 (7.9%)	24 (9.4%)	248 (9.3%)
(Missing)	0	3	11	21	341	376
Baseline amyloid status, *n* (%)						
Elevated	0	48 (45%)	358 (54%)	253 (42%)	87 (38%)	746 (46%)
Non‐elevated	0	58 (55%)	310 (46%)	352 (58%)	145 (63%)	865 (54%)
(Missing)	819	25	122	91	364	1421
Baseline ADAS‐Cog Item 13 Total Score						
Mean (SD)	18.4 (9.2)	12.4 (5.4)	16.1 (10.1)	13.1 (8.9)	14.1 (8.7)	15.5 (9.4)
(Missing)	8	1	7	10	15	41
Baseline CDR Sum of Boxes Score						
Mean (SD)	1.8 (1.8)	1.2 (0.7)	1.5 (1.9)	1.0 (1.6)	1.1 (1.6)	1.4 (1.7)
(Missing)	1	0	0	0	1	2
Baseline MMSE Score						
Mean (SD)	26.7 (2.7)	28.3 (1.5)	27.4 (2.7)	28.0 (2.5)	27.6 (2.5)	27.4 (2.6)
(Missing)	1	0	0	0	4	5

Abbreviations: ADAS‐Cog, Alzheimer's Disease Assessment Scale–Cognitive subscale; ADNI, Alzheimer's Disease Neuroimaging Initiative; APOE, apolipoprotein E; CDR, Clinical Dementia Rating; CN, cognitive normal; DEM, dementia; MCI, mild cognitive impairment; and MMSE, Mini‐Mental State Examination.

* Column‐wise percentage; *n*(%).

**TABLE 2:** A4 and LEARN: Subject characteristics by study.

Characteristic	A4 *N* = 1169^a^	LEARN *N* = 538^a^	Overall *N* = 1707^a^
Age, years			
Mean (SD)	71.9 (4.8)	70.5 (4.3)	71.5 (4.7)
Sex, *n* (%)			
Male	475 (41%)	208 (39%)	683 (40%)
Female	694 (59%)	330 (61%)	1024 (60%)
Education, years			
Mean (SD)	16.6 (2.8)	16.8 (2.6)	16.6 (2.8)
Race, *n* (%)			
American‐Indian or Alaskan native	2 (0.2%)	5 (0.9%)	7 (0.4%)
Asian	24 (2.1%)	12 (2.2%)	36 (2.1%)
Black or African‐American	28 (2.4%)	14 (2.6%)	42 (2.5%)
More than one race	8 (0.7%)	5 (0.9%)	13 (0.8%)
Unknown or not reported	7 (0.6%)	1 (0.2%)	8 (0.5%)
White	1100 (94%)	501 (93%)	1601 (94%)
Ethnicity, *n*(%)			
Hispanic or Latino	34 (2.9%)	18 (3.3%)	52 (3.0%)
Not Hispanic or Latino	1124 (96%)	516 (96%)	1640 (96%)
Unknown or not reported	11 (0.9%)	4 (0.7%)	15 (0.9%)
APOE genotype, n(%)			
E2/E2	2 (0.2%)	5 (0.9%)	7 (0.4%)
E2/E3	61 (5.2%)	66 (12%)	127 (7.4%)
E2/E4	35 (3.0%)	10 (1.9%)	45 (2.6%)
E3/E3	417 (36%)	342 (64%)	759 (45%)
E3/E4	560 (48%)	111 (21%)	671 (39%)
E4/E4	94 (8.0%)	2 (0.4%)	96 (5.6%)
(Missing)	0	2	2
Amyloid PET, centiloids			
Mean (SD)	66.1 (32.8)	4.2 (12.6)	46.6 (40.2)

Abbreviations: A4, Anti‐Amyloid Treatment in Asymptomatic Alzheimer's study; APOE, apolipoprotein E; LEARN, Longitudinal Evaluation of Amyloid Risk and Neurodegeneration.

^a^ Column‐wise percentage; *n*(%).

## 4 DISCUSSION

The development of A4LEARN and ADNIMERGE2 represents a step forward in enabling the AD research community to share and analyze data more effectively, serving as a template for additional future study packages. These packages facilitate the transition from proprietary software like SAS to open‐source tools, allowing greater flexibility and transparency in research. The shift from SAS to R reflects a broader trend in the clinical research community toward open‐source and reproducible research practices, as exemplified by the pharmaverse project.

Challenges remain, particularly in the area of data access. In our examples, data packages can be sourced using the existing data access models. This puts the onus on data users to go to different sites to obtain data. Once retrieved, the use of locally installed packages poses potential risks, which can be mitigated by using containerization and package management tools like Docker and renv^23^ for version control. A streamlined future improvement on this model might entail the use of access keys and Application Programming Interfaces (APIs) to source data directly within R.

While R packages are well‐suited for sharing rectangular data structures commonly used in statistical analyses, they are not an ideal mechanism for distributing large, complex files such as raw imaging data (e.g., DICOM or NIfTI) or full genomic datasets, which often require specialized preprocessing prior to analysis. To address this, the packages include summaries of imaging‐derived measures (e.g., hippocampal volume, cortical thickness, positron emission tomography standardized uptake value ratios [PET SUVRs]) and key genetic information such as APOE genotype, along with clinical and fluid biomarker data. This approach balances accessibility and reproducibility while avoiding the logistical and privacy challenges associated with sharing raw files.

While the packages facilitate data access, they do not prescribe pooling strategies across sites or phases. Researchers should consider potential sources of heterogeneity, such as scanner differences and protocol changes, and apply harmonization techniques or statistical adjustments appropriate for their analyses.

We envision the alzverse project expanding to include many more studies. Our plan is to continue building R data packages for studies conducted by the Alzheimer's Therapeutic Research Institute and the Alzheimer's Clinical Trial Consortium, with the hope of eventually expanding to other groups through a larger collaborative community. At some point, however, the meta‐package might become impractically large. One way to mitigate this limitation would be to allow users to select which studies to include in their personalized version of the alzverse.

## CONFLICT OF INTEREST STATEMENT

M.C.D. is a consultant to F. Hoffmann‐La Roche Ltd, and his spouse is a full‐time employee of Johnson & Johnson. P.S.A. has research grants from NIH, the Alzheimer's Association, Janssen, Lilly, and Eisai, and consults with Merck, Roche, Genen‐tech, Abbvie, Biogen, and ImmunoBrain Checkpoint. M.D., G.J.‐M., and O.L. report research grants from NIA, Eisai, Eli Lilly, Alzheimer's Association, Gates Ventures, and the Epstein Family Foundation. Other authors report no relevant competing interests. Author disclosures are available in the Supporting Information.

## CONSENT STATEMENT

All human subjects provided informed consent.

## Supporting information



Supporting Information

Supporting Information

## Footnotes

1 Based on a Google Scholar search of “ADNIMERGE” AND “package”
