# Supplementary material for: Alzheimer's clinical research data via R packages: The alzverse
Source: Alzheimers Dement. 2026 Jan 29;22(2):e71152. doi: 10.1002/alz.71152 (PMC12854935; doi:10.1002/alz.71152)
Supplement: Supplementary file 2 — Supporting Information [file ALZ-22-e71152-s002.docx]

## Appendix

### Data Availability

Data is available from:

- A4LEARN: [A4StudyData.org](file:///var/folders/b0/934tl8yj1v39y6j436kcwn_00000gr/T/Rtmp3BFdam/preview-1535730d2ed79.dir/A4StudyData.org)
- ADNIMERGE2: [loni.usc.edu](file:///var/folders/b0/934tl8yj1v39y6j436kcwn_00000gr/T/Rtmp3BFdam/preview-1535730d2ed79.dir/loni.usc.edu)

Documentation is available from

- A4LEARN: [atri-biostats.github.io/A4LEARN/](https://atri-biostats.github.io/A4LEARN)
- ADNIMERGE2: [atri-biostats.github.io/ADNIMERGE2](https://atri-biostats.github.io/ADNIMERGE2)

### Code Availability

R code is available for download from the following repositories:

- alzverse: [github.com/atri-biostats/alzverse](https://github.com/atri-biostats/alzverse)
- A4LEARN: [github.com/atri-biostats/A4LEARN](https://github.com/atri-biostats/A4LEARN)
- ADNIMERGE2: [github.com/atri-biostats/ADNIMERGE2](https://github.com/atri-biostats/ADNIMERGE2)

#### R Code for the ADNIMERGE2 Example

tbl_summary(
 data = ADNIMERGE2::ADSL %>% filter(ENRLFL %in% "Y"),
 by = ORIGPROT,
 include = c(AGE, SEX, EDUC, RACE, ETHNIC, DX, APOE, AMYSTAT,
 ADASTT13, CDRSB, MMSCORE),
 type = all_continuous() ~ "continuous2",
 statistic = list(
 all_continuous() ~ "{mean} ({sd})",
 all_categorical() ~ "{n} ({p}%)"),
 digits = all_continuous() ~ 1,
 percent = "column",
 missing_text = "(Missing)") %>%
 add_overall(last = TRUE) %>%
 add_stat_label(label = all_continuous2() ~ "Mean (SD)") %>%
 modify_footnote_header(
 footnote = "Column-wise percentage; n (%)",
 columns = all_stat_cols(),
 replace = TRUE) %>%
 bold_labels()

# Individual profile (spaghetti) plot
ADNIMERGE2::ADQS %>%
 # Enrolled participant
 filter(ENRLFL %in% "Y") %>%
 # ADAS-cog item-13 total score
 filter(PARAMCD %in% "ADASTT13") %>%
 mutate(Years = convert_number_days(ADY, unit = 'year')) %>%
 filter(!if_any(all_of(c("Years", "DX", "AVAL")), ~ is.na(.x))) %>%
ggplot(aes(x = Years, y = AVAL, group = USUBJID, color = DX)) +
 geom_line(alpha = 0.25) +
 scale_color_manual(values = c("#73C186", "#F2B974", "#DF957C", "#999999")) +
 labs(
 y = "ADAS-cog13 Total Score",
 x = "Years since baseline visit",
 color = "Baseline Diagnostics Status") +
 theme(legend.position = "bottom") +
 guides(colour = guide_legend(override.aes = list(alpha = 1)))

#### R Code for the A4LEARN Example

tbl_summary(
 data = A4LEARN::SUBJINFO %>% filter(SUBSTUDY %in% c('A4', 'LEARN')),
 by = SUBSTUDY,
 include = c(AGEYR, SEX, EDCCNTU, RACE, ETHNIC, APOEGN, AMYLCENT),
 label = list(AGEYR = "Age, years", SEX = 'Sex',
 EDCCNTU = 'Education, years', RACE = 'Race', ETHNIC = 'Ethnicity',
 APOEGN = 'APOE genotype', AMYLCENT = 'Amyloid PET, Centiloids'),
 type = all_continuous() ~ "continuous2",
 statistic = list(
 all_continuous() ~ "{mean} ({sd})",
 all_categorical() ~ "{n} ({p}%)"),
 digits = all_continuous() ~ 1,
 percent = "column",
 missing_text = "(Missing)") %>%
 add_overall(last = TRUE) %>%
 add_stat_label(label = all_continuous2() ~ "Mean (SD)") %>%
 modify_footnote_header(
 footnote = "Column-wise percentage; n (%)",
 columns = all_stat_cols(),
 replace = TRUE
 ) %>%
 bold_labels()

#### R Code for the alzverse Example

alzverse::ADQS %>%
 filter(DX == 'CN' | STUDYID %in% c('A4', 'LEARN'),
 PARAMCD %in% c('MMSCORE', 'MMSE')) %>%
 mutate(Years = convert_number_days(ADY, unit = 'year')) %>%
ggplot(aes(x = Years, y = AVAL, color = STUDYID)) +
 geom_line(aes(group = USUBJID), alpha = 0.25) +
 labs(
 y = "MMSE Total Score",
 x = "Years since baseline visit",
 color = "Study") +
 theme(legend.position = "bottom") +
 guides(colour = guide_legend(override.aes = list(alpha = 1)))
